# Understanding the Issue of Alcoholism in the British Sikh Punjabi Community. Based on This, How Can the Medical School Curriculum Be Improved So Clinicians Can Better Meet the Needs of the British Sikh Punjabi Community and Diverse Communities in General?

**DOI:** 10.1192/bjo.2024.188

**Published:** 2024-08-01

**Authors:** Ankita Kochhar

**Affiliations:** Queen Mary University of London, London, United Kingdom

## Abstract

**Aims:**

1. Identify the factors that shape alcohol consumption and accessing support for excessive alcohol consumption in the BSP community.
2. Establish the current provision of alcohol-related education in the UK medical school curriculum and analyse if this is suitable to address alcoholism in the BSP community.
3. Provide recommendations to be made to the curriculum to help medical students approach the issue of alcoholism in specific communities in a culturally competent manner.

**Methods:**

Two narrative literature reviews were conducted. 37 studies were included. The first search underwent thematic analysis with reference to a Public Health England framework, and the second underwent inductive thematic analysis. Subsequently, the results from both searches were compared to produce appropriate recommendations.

**Results:**

Factors Influencing Alcoholism in the BSP Community

- Experiences of racial discrimination result in psychological distress, and the need to acculturate to decrease this risk.
- Loneliness, mainstream Punjabi music, and a decreased self-reported importance of religion.
- The role of masculinity was emphasized, with both those who abstained and those who drink viewed as masculine.

Alcohol-related Education and Medical School

- Alcohol use has increased among UK medical students.
- The drinking habits of medical students are crucial to their own health, their clinical practice, and indirectly as role models in society for acceptable lifestyle behaviours.
- Approximately 14 hours are dedicated to alcohol and drug-misuse teaching over the 5-year medical school degree.
- Lack of alcohol-related-policies at UK medical schools.
- Doctors’ negative attitudes towards patients with AUD were frequently reported.
- Medical students are eager to learn about AUD.

Recommendations for the Medical School Curriculum:

- Development of a comprehensive and supportive alcohol-related policy.
- Pre-clinical teaching: seminars with an individual who has recovered from an AUD.
- Clinical stage teaching: encourage students to write and present cases of patients with AUD.
- Encourage the use of non-judgemental labels.
- Lectures including speakers from voluntary AUD services.
- Encourage Alcoholics Anonymous attendance for students.

**Conclusion:**

Overall, the BSP population fail to access treatment services due to fear of shame and stigma. Medical schools have immense potential to make changes to their alcohol-related education to ensure that future doctors provide holistic care, leading to earlier detection and management of alcohol-use disorders. Recommendations were made with the intention of providing culturally competent services.

